# Health Tract No. 325

**Published:** 1868-07

**Authors:** 


HEALTH TRACT No. 325.

### THE EGO.

He is not more the world's benefactor who causes two blades of grass to grow, where but one grew before, than he who takingly expresses a great practical truth. Oliver Wendel Holmes has well put it in the first Atlantic Monthly Almanack "one cannot gather some of the best fruits of life without climbing out to the end of the slender branches of 'The Ego,'" meaning that many a truth is most valuable and is most impressively conveyed, by the person being able to say, "I" did so and so ; this form of communication has a tangibility about it and a force, which the sentimental expression of the very same idea can never have, for there is a kind of demonstration about it, a proof, which gives it power ; hence under certain restrictions, it is not unprofitable to write about ourselves ; only as to health, it is not safe to generalize too quickly ; and yet we are constantly running into grave errors in this, direction, errors which imperil health and even life itself. If a man says, "I was very ill, did thus and was cured," it would not be safe for another who was ill to do the same thing, unless all the circumstances were similar. The fabled Donkey, on the very point of exhaustion on a hot summers day from a heavy burden of salt, came to a river, boldly plunged in, and found his load greatly diminished when he emerged from the other shore ; another hearing this, and afterwards almost crushed with a load of wool, tried the experiment, and was carried to the bottom. Hence it is in almost every man's experience, that what cured some one else, a great deal worse than himself, failed to impart to him the slightest possible benefit. If a man is afraid to attempt to repair his watch' when out of order, he should certainly hesitate to tinker with the infinitely more delicate machinery of his own body. They are wisest, who when really ailing, do nothing on their own responsibility, but promptly consult a physician.

